# Effects of neutron radiation generated in deep space-like environments on food resources

**DOI:** 10.1038/s41598-023-38990-1

**Published:** 2023-08-01

**Authors:** Machiko Hatsuda, Hiroaki Kawasaki, Ayako Shigenaga, Atsushi Taketani, Takaoki Takanashi, Yasuo Wakabayashi, Yoshie Otake, Yayoi Kamata, Akari Ichinose, Honoka Nishioka, Hayato Kimura, Yumi Koganei, Shiomi Komoriya, Miyu Sakai, Yui Hamano, Maria Yoshida, Fumiyuki Yamakura

**Affiliations:** 1 Faculty of Health Science, Juntendo University, Tokyo, 113-0034 Japan; 2 Faculty of Food and Health Sciences, Showa Women’s University, Tokyo, 154-8533 Japan; 3 Institute of Health and Sports Science and Medicine, Juntendo University, Chiba, 270-1695 Japan; 4 RIKEN Center for Advanced Photonics, RIKEN, Saitama, 351-0198 Japan; 5 Institute for Environmental and Gender-Specific Medicine, Juntendo University, Chiba, 279-0023 Japan

**Keywords:** Proteins, Biophysical chemistry, Space physics, Risk factors, Experimental nuclear physics, Chemical physics

## Abstract

The impact of deep space cosmic rays on food resources is as important as the risks of cosmic rays to the human body. This study demonstrates the potential for neutrons as secondary radiation in deep space spacecraft to cause meat activation and oxidative modification of proteins and lipids. We conducted a series of experiments such as the neutron irradiation experiment, the radioactivation analysis and the biochemical analysis. Neutrons with energies from 1 to 5 MeV with doses from 0.01 Gy to 4 Gy were irradiated by the RIKEN accelerated-driven neutron source (RANS). Radioactive nuclei, ^24^Na, ^42^K, and ^38^Cl, were detected in the neutron-irradiated meat. The modification products of the proteins by oxidative nitration, 6-nitrotryptophan (6NO_2_Trp), and by a lipid peroxidation, 4-hydroxy-2-nonenal (4-HNE), were detected in several proteins with neutron dose dependent. The proteome analysis showed that many oxidative modifications were detected in actin and myosin which are major proteins of myofibrils. This study is of crucial importance not only as risk factors for human space exploration, but also as fundamental effects of radiation on the components of the human body.

## Introduction

Cosmic rays in deep space such as Moon and Mars include energetic particles with various atomic numbers^1–11^. To protect the human body from those primary cosmic rays, the walls of spacecraft have to be thickened. The interaction between those walls and primary cosmic rays then generates secondary particles^6–11^. Secondary particles are 30% each of γ’s, neutrons and protons with the other population of pions and α particles^6^. For the shielding of 15 g/cm^2^ of aluminum, the flux of the neutrons is significantly large inside spacecraft^7^. In reference^7^, neutrons at energies lower than 100 MeV overwhelmingly contribute to the flux in spacecraft in deep space. For example at 1 MeV, the neutron flux is the largest, the proton flux is 10^–3^ times the neutron flux, the α particle flux is 10^–4^ times it, and other particles with Z > 2 are much smaller than them. Neutron flux decreases as neutron energy increases and is overtaken by proton flux at about 100 MeV. Mars mission exposure for 420 days free space and 500 days Mars surface is estimated 0.45 Gy^2,7^. The deep space environment also includes inside containers to store food resources in lunar and Martian settlements, where longer storage periods have a greater impact.

**Figure 1:**
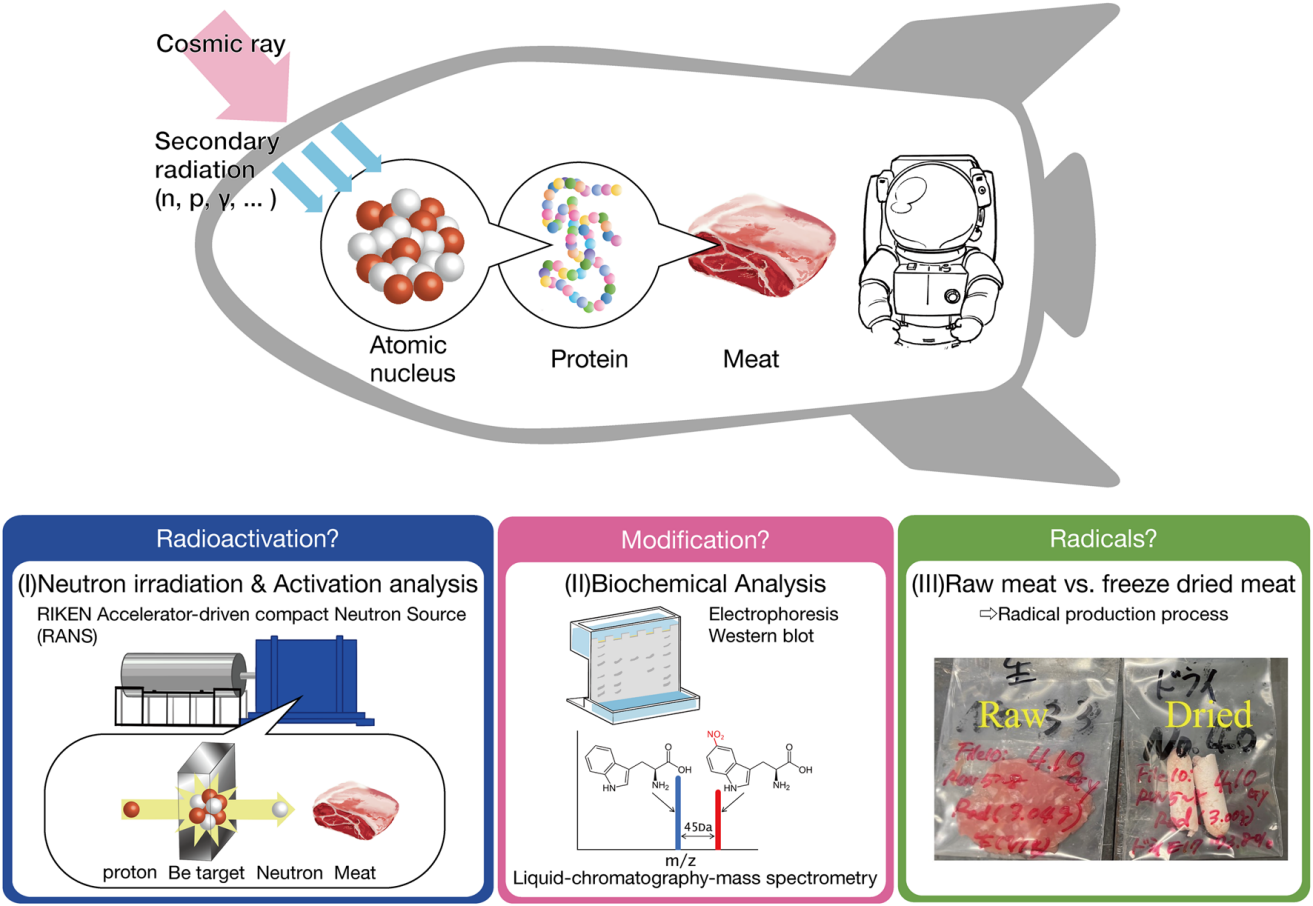
Multiscale phenomena as the effects of neutron irradiation on meat. (I) Neutron irradiation on pork shoulder meat performed by RANS and radioactivation analysis to detect radioactivation of meat. (II) Biochemical analysis such as electrophoresis, Western blotting, and liquid chromatography-mass spectroscopy to detect modification of proteins and lipids. (III) A comparative experiment of raw meat and freeze-dried meat to clarify formation mechanism of radicals.

Studies on the irradiation of food resources with photons and electrons had been carried out for the purpose of sterilization and prevention of germination^12^, and their chemical toxicity had also been investigated^13^. Neutrons are now used in airport inspections and research into the effects of neutron radiation on food has begun^14^. In contrast to the effects of the irradiation of γ’s and electrons, the neutron irradiation involves completely different interactions such as the neutron capture and the nuclear reactions producing protons and α particles. So the effects of neutron irradiation on food resources cover a wide range of phenomena at different scales (Fig. 1). At first neutrons collide with atomic nuclei, then nuclear reactions generate radioactive nuclei. Next, modifications of protein and lipid occur through biochemical reactions at the protein scale.

Sufficient amounts of essential amino acids cannot be synthesized in the human body and must therefore be obtained from food. In general oxidative modifications of proteins induced by reactive oxygen species and reactive nitrogen species can impair protein function. Nitric oxide (NO) reacts with superoxide radicals (O_2_^−^) in vivo to form highly toxic peroxynitrite (ONOO^-^), which is highly reactive and modifies proteins and other biomolecules causing damage to cells and organs. Tryptophan which is one of the essential amino acids reacts with peroxynitrite to form 6-nitrotryptophan (6NO_2_Trp)^15^. 6NO_2_Trp has been detected in small animal models of atopic dermatitis, spontaneous hypertension, and diabetes^15–24^. A wide variety of degradation products of lipids are produced by lipid peroxidation in food and in vivo under oxidative conditions. 4-hydroxy-2-nonenal (4-HNE) is the most representative degradative product of lipid by the oxidation. These compounds are produced in the cells, tissues of living organisms and food. They are considered causative agents of many diseases, including various types of cancer^25^.

In this study, we have conducted a series of experiments^26^ from the neutron irradiation experiment to the radioactivation and the biochemical analysis whose experimental setup is given in Fig. 1: (I) Neutron irradiation on pork shoulder meat was performed by RIKEN accelerator-driven neutron source (RANS)^27–31^. The radioactivation analysis includes measurement of the γ-ray spectra by the germanium (Ge) detector and the radiation measurement by the Geiger–Müller (GM) counter. (II) The biochemical analysis includes electrophoresis (SDS-PAGE), Western blotting, and proteome analysis by liquid chromatography-mass spectroscopy (LC–MS/MS). Understanding the effects of neutron irradiation on meat requires a cross-disciplinary understanding of biochemical reactions, from the physics of nuclear reactions to the oxidative modification of proteins and lipids. (III) In order to clarify the mechanism of radical formation necessary for oxidative modification to occur we performed the comparative experiment of raw meat and freeze-dried meat which reveals the role of water.

## Results

Results are reported in the order shown in Fig. 1 as (I) neutron irradiation and radioactivation analysis, (II) biochemical analysis, and (III) a comparative experiment of raw meat and freeze-dried meat.

### Neutron irradiation

The fast neutron irradiation on meat was performed by RANS as shown in (I) of Fig. 1. The beryllium (Be) target in the target station (blue box) is bombarded by the 7 MeV proton accelerated in the RF linear accelerator (gray tube). The nuclear reaction ^9^Be(p,n)^9^B produces fast neutrons impinging on the sample with energy about 1 to 5 MeV mainly, as a consequence of the two-body nuclear process and of possible secondary interactions of neutrons with the material surrounding the sample. Thermal neutrons of 20 meV to 200 meV are also produced, but the thermal neutron peak is small enough compared with the fast neutron peak in a situation where no moderator is placed.

The neutron dose is controlled by the proton current and the irradiation time ${t}_{i}$. The neutron dose was numerically simulated by the radiation transport code PHITS^32^. The irradiated neutron numbers were counted through the indium (In) radioactivation (activation) analysis. Indium foils with an area of 1.0 × 1.0 cm^2^ and a thickness of 125 μm were placed with the meat samples under neutron irradiation. The number of γ-ray of the radioisotope of indium, ^115m^In produced by the neutron irradiation on ^115^In, was counted by the germanium (Ge) detector. In Table 1, neutron doses calculated by PHITS, parameters of RANS experiments and neutron numbers obtained by the indium activation analysis for (0.01, 0.03, 0.1, 2.0, 3.0) Gy are listed. The parameters of RANS experiments are as follows: the proton current and the neutron irradiation time ${t}_{i}$, the mass of indium foil $W$, the cooling time ${t}_{c}$, the measurement duration time of the γ-ray of the radioisotope of indium ${t}_{m}$, and the counted number of γ-rays ${C}_{\gamma }$. The neutron flux $\Phi$ and the total number of irradiated neutrons $N$ are obtained by using the Eq. (3) in Method.

**Table 1:** RANS neutron beam: proton currents, irradiation times, neutron fluxes and numbers of neutrons. The uncertainty of the neutron number in the last column is evaluated as the standard error caused by the area of the peaks of *γ*’s and the detection efficiency 2% of *ε*.

Dose by PHITS (Gy)	Proton current (μA)	Irradiation time	Mass of In foil	Detection efficiency	Cooling time	Measurement time	Counts of γ	Neutron flux	Number of neutrons
		${t}_{i}$(s)	$W$(mg)	$\varepsilon$	${t }_{c}$(s)	${t}_{m}$(s)	${C}_{\gamma }$(n)	$\Phi$(10^8^ n/s/cm^2^)	$N$(10^8^ n/cm^2^)
0.01	5.72	30	104.09	0.015	459	1008	280	3.2	96 ± 5
					4699	3600	871		
					8300	1725	369		
0.03	5.69	90	105.94	0.015	545	1219	1322	3.9	351 ± 15
					18,197	1800	878		
					19,997	1800	884		
					21,798	1800	735		
					23,599	1800	743		
					25,400	1800	687		
					27,201	1800	653		
					29,002	1392	431		
0.10	5.71	301	104.25	0.008	687	1226	1557	2.7	798 ± 26
1.00	33.40	421	104.87	0.005	572	1598	9977	16.8	7157 ± 160
					2207	1800	11,357		
					4008	1800	10,419		
					5809	1603	8688		
2.32	34.41	984	104.14	0.005	29,390	3600	17,947	19.8	19,680 ± 420
3.00	33.85	1270	97.73	0.005	18,260	3600	31,896	18.1	22,860 ± 534
					21,861	3600	27,426		
					25,461	3600	23,252		
					29,063	3600	20,017		
					32,664	3600	17,144		
					36,265	3600	14,804		
					39,866	3600	12,869		
					43,467	3600	10,765		
					47,068	3600	9186		
					50,669	3600	7940		
					54,270	545	1100		

**Figure 2:**
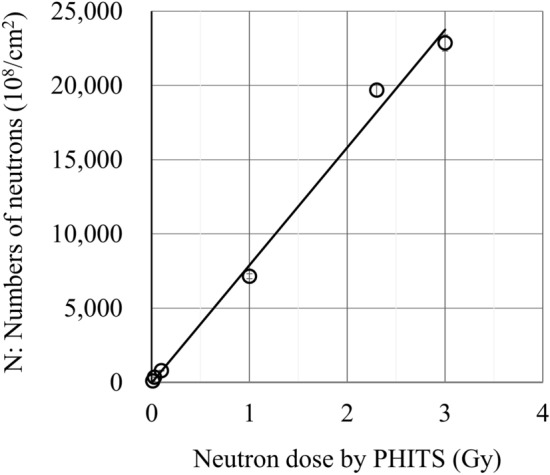
Numbers of neutrons measured by the ^115^In activation analysis and neutron doses calculated by PHITS.

Figure 2 shows the positive correlation between numbers of irradiated neutrons measured by the indium (In) activation analysis and neutron doses calculated by PHITS. This confirms that the numbers of neutrons produced by RANS with these parameters realize the linear dependence on neutron doses.

There exists the background of thermal neutrons and γ-rays produced by the cooling water and the shield inside the target station. The γ-ray dose was simulated by PHITS as almost 1/10 of the neutron dose.

### Activation analysis

**Figure 3:**
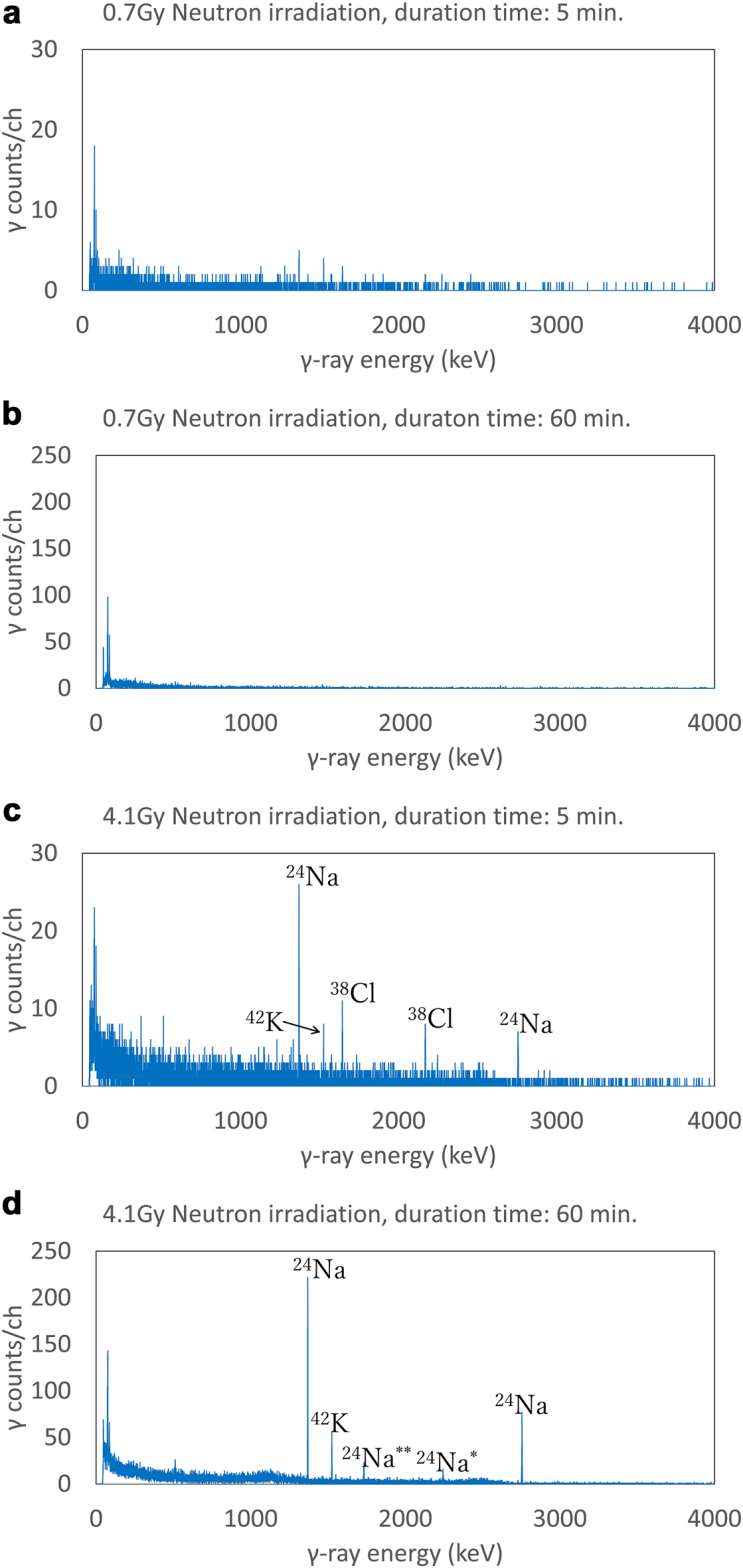
γ-ray spectra of neutron-irradiated meats with neutron doses and measurement duration times: (**a**) 0.7 Gy, 5 min, (**b**) 0.7 Gy, 60 min, (**c**) 4.1 Gy, 5 min, (**d**) 4.1 Gy, 60 min. Detected radioactive nuclei with γ-ray energies are the followings: ^24^Na with 1369 keV and 2754 keV, ^42^K with 1525 keV, ^38^Cl with 1642 keV and 2168 keV, ^24^Na* single escape peak with 2243 keV, ^24^Na** double escape peak with 1732 keV.

We show that neutron irradiation causes the radioactivation of meat. The γ-ray spectra of neutron-irradiated meat samples are given in Fig. 3. To find short-lived radioactive nuclei the 5 min measurements were taken in addition to the 60 min measurements which are enough to detect peaks with statistical significance. In Fig. 3a,b the γ-ray spectra of 0.7 Gy neutron dose for both 5 min and 60 min measurements, no significant peaks due to radioactive nuclei from meat samples are detected. Peaks at the γ-ray energy less than 50 keV are background caused by the lead that makes up the shielding walls of the Ge detector. In Fig. 3c the γ-ray spectrum of 4.1 Gy neutron dose for 5 min measurement, peaks are found as the following elements with γ-ray energies and the half-life^33–36^ in parentheses as (E_γ_;τ): ^24^Na (1368.63 keV, 2754.01 keV; 14.99 h), ^38^Cl (1642.43 keV, 2167.54 keV; 37.24 m) and ^42^K (1524.60 keV; 12.36 h). In Fig. 3d the γ-ray spectrum with 4.1 Gy neutron dose for 60 min measurement, peaks are found as the following elements: ^24^Na (1368.63 keV, 2754.01 keV; 14.99 h), the single escape peak of ^24^Na (2754.01 $-$ 511.00 = 2243.01 keV), the double escape peak of ^24^Na (2754.01 $-$ 2 × 511.00 = 1732.01 keV) and ^42^K (1524.60 keV; 12.36 h).

These radioactive nuclei are generated by the following nuclear reactions^33–35^:

$$\begin{gathered} {\text{Neutron capture}}:{^{23} \text{Na}} + {\text{n}} \to{^{24} \text{Na}} + \gamma \hfill \\ {\text{Neutron capture}}:{^{37}\text{Cl}} + {\text{n}} \to{^{38}\text{Cl}} + \gamma \hfill \\ {\text{Neutron capture}}:{^{41}\text{K}} + {\text{n}} \to^{^{42}\text{K}} + \gamma \hfill \\ \left( {{\text{n}},{\text{p}}} \right){\text{ reaction}}:{^{42}\text{Ca}} + {\text{n}} \to{^{42}\text{K}} + {\text{p}} \hfill \\ ({\text{n}},\alpha ){\text{ reaction}}:{^{41}\text{K}} + {\text{n}} \to{^{38}\text{Cl}} + \alpha \hfill \\ \end{gathered}$$

The nuclear reaction, ^41^K(n,α)^38^Cl, occurs for the neutron energy above 2 MeV, and ^42^Ca(n,p)^42^K occurs for the neutron energy above 2.5 MeV. The mass composition ratios of elements of this meat sample by the ICP mass spectrometry (Supplement Table 2) were the following with the isotope abundance ratio θ in parentheses: Na (^23^Na, θ = 1.0000) 460 ppm, Ca (^40^Ca, θ = 0.9694; ^42^Ca, θ = 0.0065) 41 ppm, K (^39^K, θ = 0.9326; ^41^K, θ = 0.0673) 0.37%. The mass composition ratio of Cl was not measured by the ICP mass spectrometry. These trace elements in meat are found to be involved in radioactivation.

### Biochemical analysis—electrophoresis and Western Blotting (WB)

**Figure 4:**
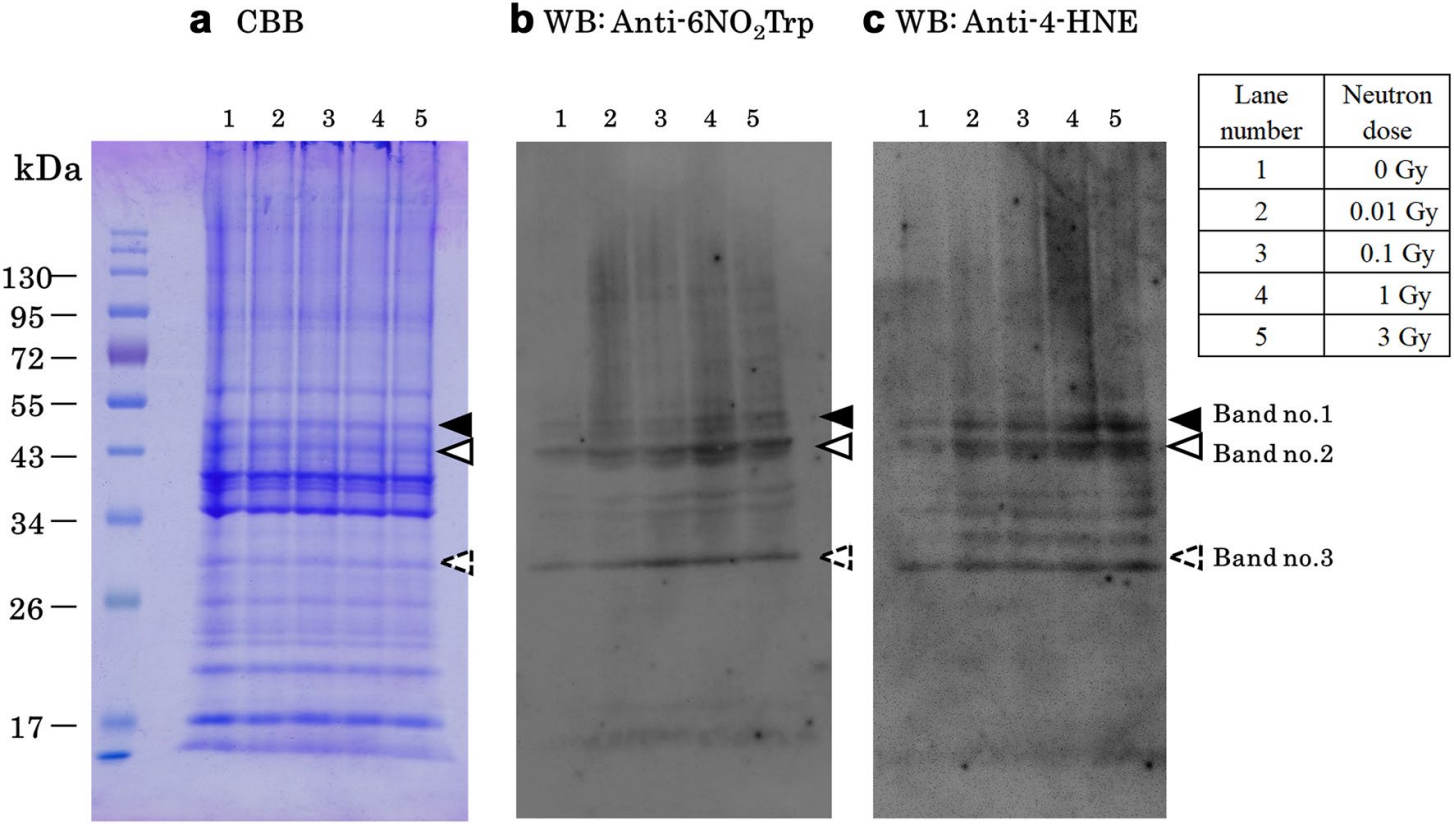
Neutron dose dependence of modification signals. Band no.1–3 corresponds to 50 kDa, 43 kDa, and 30 kDa respectively. (**a**) The electrophoresis of the neutron-irradiated meat protein with the CBB stained. (**b**) Detection of the nitrative modification of tryptophan (6NO_2_Trp) in WB with antibody. (**c**) Detection of the oxidative modification of lipid (lipid peroxidation) containing polyunsaturated ω6 acyl groups (4-HNE) in WB with antibody. (Supplementary Figs. 1, 2).

**Figure 5:**
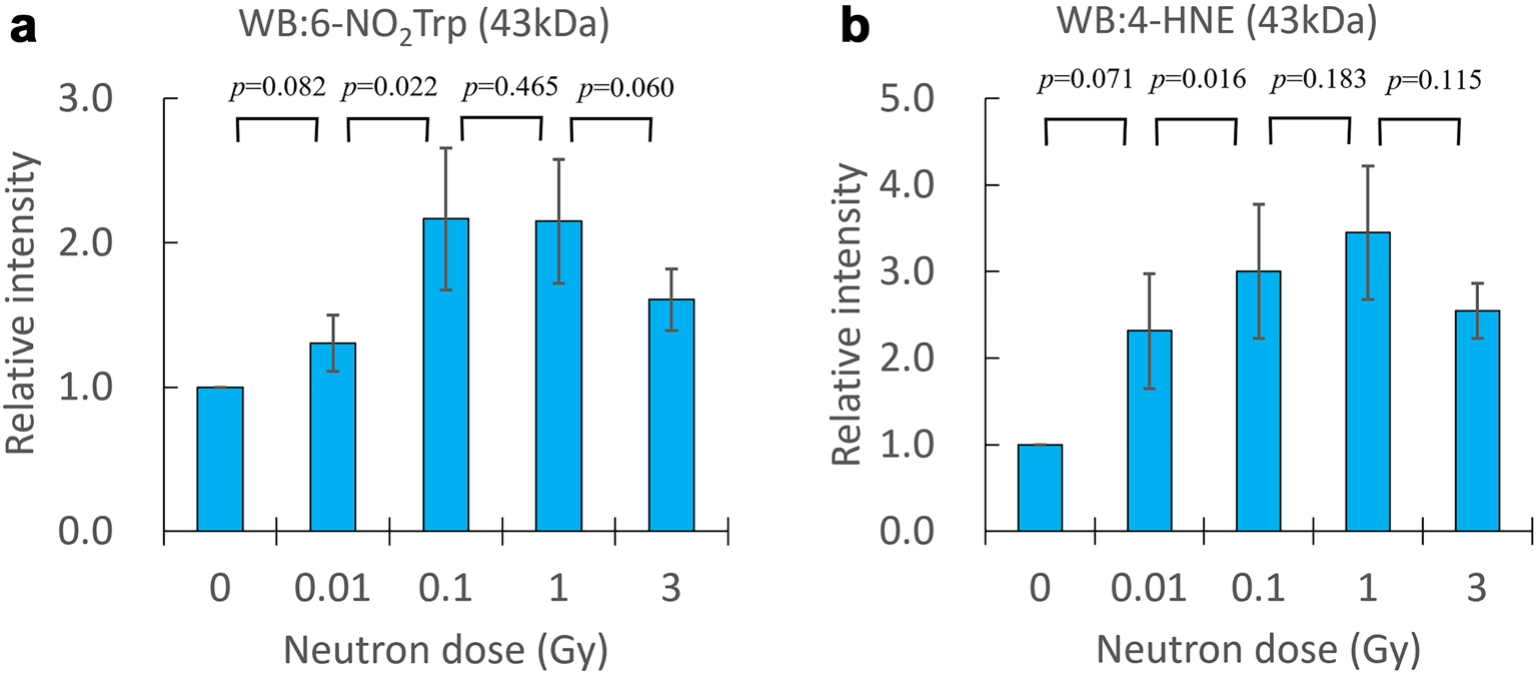
Neutron dose dependence of the relative intensity of modification. (**a**) 6NO_2_Trp and (**b**) 4-HNE in band no.2 of 43 kDa. The relative intensity and the error bar represent the average and the standard error of the mean (SEM) from the average of seven separate experiments for (**a**) and four experiments for (**b**). Relative signal intensities are normalized with the 0 Gy signals.

We show that the neutron irradiation causes oxidative modification of proteins and lipids. The correlation between the modification of proteins and lipids and the neutron dose is presented for the first time in Figs. 4 and 5. The electrophoresis of the neutron-irradiated meat proteins with the Coomassie-Brilliant Blue (CBB) stained shows that no significant cleavage or aggregation of proteins was observed (Fig. 4a). The nitrative modification of tryptophan, 6NO_2_Trp, in the proteins around 50 kDa, 43 kDa, and 30 kDa (band no. 1, 2 and 3) were detected by WB with anti-nitrotryptophan antibody (Fig. 4b). 4-HNE, which is one of the oxidative products of polyunsaturated lipid, conjugated proteins around 50 kDa, 43 kDa, and 30 kDa were detected by WB with anti-4-HNE antibody (Fig. 4c). Signals around 50 kDa, 43 kDa, and 30 kDa from the neutron-irradiated samples (lane no.2–5) are more intense than signals from the unirradiated sample (lane no.1) (Fig. 4b,c). In Fig. 5 the signal intensities are increased depending on the neutron irradiation dose. However around 1 Gy neutron irradiation, the modification signals saturate or start to decrease (Fig. 5a,b). This increase in semi-quantitative analysis of the positive signals for the anti-6-NO_2_Trp antibody and anti-4-HNE antibody in band no.2 (Band 2) of 43 kDa is shown in each of the samples. The protein bands corresponding to band no.1–3 in the CBB stained gels (Fig. 4a) are cut out, digested with trypsin, and subjected to LC–MS/MS analysis. Since the LC–MS/MS analysis detects many modified actine, Band 2 of 43 kDa actine is selected to perform the semi-quantitative analysis in Fig. 5.

### Biochemical analysis—proteome analysis

Mass spectrometry can clarify the species of nitrated protein, and various information such as the positions of modified residues that Western blotting cannot clarify. We have identified the modified proteins in neutron-irradiated meat samples as shown in Table 2. The nitrative modification of tryptophan (6NO_2_Trp) is denoted as no_2_-W and the oxidative modification of lysine and cysteine (4-HNE attached Lys and Cys) are denoted as hne-K and hne-C respectively. The presence of many oxidative modifications in actin and myosin which are the major proteins that make up myofibrils. Various oxidative modifications were detected in myofibrillar component proteins, cytoskeleton-related proteins, enzymes related to glycolytic system. We successfully identified the positions of nitrated tryptophan residue (nitration causes mass increase of 45 Da) and 4-HNE conjugated residues (target residues were lysine, histidine and cysteine. Addition of 4-HNE causes mass increase of 156 Da) for several proteins by LC–MS/MS analysis. Several tryptic peptides derived from 14 different proteins, which are beta-enolase, triosephosphate isomerase, pyruvate dehydrogenase E1 component subunit alpha somatic form mitochondrial (Fragments), phosphoglycerate kinase 1, myosin-1, trypsin, perilipin-2, Inward rectifier potassium channel 2, thyroxine-binding globulin in Band 1, actin cytoplasmic1, actin alpha skeletal muscle, creatine kinase M-type, phosphoglycerate kinase 1, myosin-1, triosephosphate isomerase in Band 2, adenylyl cyclase-associated protein 1 (Fragments), myosin-1, triosephosphate isomerase in Band 3, are found to include nitrated tryptophan residues or 4-NHE attached residues with significant ion score levels (*p* < 0.05) by MASCOT search (Table 2). Since the mass of 4-HNE and the mass of arginine residue are equal, it is possible that the insertion of arginine residue in peptide sequence due to mutation may be regarded as the addition of 4-HNE.

**Table 2:** Proteome analysis by LC–MS/MS. no_2_-W, hne-K, and hne-C indicates NO_2_-Trpresidue, 4-HNE-attached Lys and Cys residues. W(Trp, tryptophan) and K(Lys, lysine) are essential amino acids while C(Cys,cysteine) is a non-essential amino acid. All peptides cleared p < 0.05 of ion scores by the Mascot search.

Band no	Identified protein	Modified peptide sequence	Acc. No
1	Beta-enolase	K.LAQSNG**no**_**2**_**-W**GVMDVAASEFYR.N	Q1KYT0
		R.IAQAVE**hne-K**.K	
	Triosephosphate isomerase	K.VVLAYEPVWAIGTGKTATPQQAQEVHE**hne-K**.L	Q29371
	Pyruvate dehydrogenase E1 component subunit alpha, somatic form, mitochondrial (Fragments)	R.SG**hne-K**GPILMELQTYR.Y	P29804
	Phosphoglycerate kinase 1	K.KAGGFLM**hne-K**.K	Q7SIB7
	Myosin-1	R.VRELEGEVESEQ**hne-K**.R	Q9TV61
	Trypsin	K.DSCQGDSGGPVVCNGQLQGIVS**no**_**2**_**-W**GYGCAQK.N	P00761
	Perilipin-2	K.SL**hne-C**EMAEK.G	Q4PLW0
	Inward rectifier potassium channel 2	R.CVTDE**hne-C**PIAVFMIVFQSIVGCIIDAFIGAVMA**hne-K**MA**hne-K**.P	O18839
	Thyroxyne-binding globulin	K.NALALFVLPNEGQME**no**_**2**_**-W**VEGAMSSK.I	Q9TT35
2	Actin, cytoplasmic1	K.YPIEHGIVTN**no**_**2**_**-W**DDMIEK.I	Q6QAQ1
		K.DSYVGDEAQS**hne-K**.R	
	Actin, alpha skeletal muscle	K.DSYVGDEAQS**hne-K**.R	P68137
	Creatine kinase M-type	K.LSVEALNSLTGEF**hne-K**.G	Q5XLD3
	Phosphoglycerate kinase 1	R.VDFNVPM**hne-K**NNQITNNQR.I	Q7SIB7
	Myosin-1	R.VRELEGEVESEQ**hne-K**.R	Q9TV61
	Triosephosphate isomerase	K.VVLAYEPVWAIGTGKTATPQQAQEVHE**hne-K**.L	Q29371
3	Adenylyl cyclase-associated protein 1 (Fragments)	K.HI**no**_**2**_**-W**NGQK.-	P40125
	Myosin-1	R.VRELEGEVESEQ**hne-K**.R	Q9TV61
	Triosephosphate isomerase	K.VVLAYEPVWAIGTGKTATPQQAQEVHE**hne-K**.L	Q29371

#### Abbreviations and biochemical terminologies

“SDS-PAGE (sodium dodecyl sulfate–polyacrylamide gel electrophoresis)” is a discontinuous electrophoresis system commonly used as a method of separating proteins with molecular weights of 5 to 250 kDa.“Da(dalton)” is a unified atomic mass unit (non-SI unit) which is defined as 1/12 of the mass of C-12. “CBB(Coomassie-Brilliant Blue)” is the name of coloring used for staining proteins in analytical biochemistry. “Proteome analysis” is the separation, identification and quantification of the entire protein complement expressed by a genome, a cell or a tissue. “Electrophoresis” is a method to separate molecules depending on their charges and masses in the electric field. “ICP (inductively　coupled plasma)” is a type of mass spectrometry that uses an inductively coupled plasma to ionize the sample. “Western blotting” is a method to identify specific proteins from a complex mixture of proteins extracted from cells, that uses three elements to accomplish this task. (1) Separation by using electrophoresis, (2) transfer to a solid support (PVDF membrane), and (3) marking target protein using a proper primary and secondary antibody to visualize. “CHAPS” is a zwitterionic surfactant used in the laboratory to solubilize biological macromolecules such as proteins.“PVDF (polyvinylidene difluoride)” is a highly non-reactive thermoplastic fluoropolymerused as a transfer membrane of proteins from the polyacrylamide gel.

### Comparison of dried meat sample and raw meat sample

The radiation counts, including β-rays and γ-rays, of the raw meat sample and the freeze-dried meat sample were measured by the GM counter (Table 3). In experiment 1, the radiation counts of dried meat sample are larger than the radiation counts of raw meat sample. Shielding effect of β-ray by water in meat was also examined as follows. A quantity of water equal to the mass of evaporated water was added to the dried meat sample, then the radiation count was reduced to close to the one of raw meat. This result implies that water shields the β-ray radiation. In order to clarify the role of water in meat activation and the role of water in radiation emission from meat, another validation is necessary.

**Table 3:** Radiation counts of raw meat and dried meat samples were measured by the GM counter. t_c_ is the cooling time (hour) which is the time from the end of irradiation to the start of measurement. R_dry_ is the mass ratio before and after freeze-drying of the meat sample. (a) In experiment 1, the raw meat sample and the dried meat sample were different pork shoulder meats. (b) In experiment 2, the raw meat sample and the dried meat sample were the same pork shoulder meat so the raw meat sample was kept at −80 ℃ for a night (Supplemental Table 1).

a. Experiment 1 (4.1Gy)				
Background	(55.8 ± 10.8) cpm	t_c_ (h)	Mass of sample	R_dry_
Raw meat	(2.43 ± 0.06) kcpm	1:50	3.00 g	
Dried meat	(8.33 ± 0.10) kcpm	1:51	3.00 × (1–0.738)g	73.80%

b. Experiment 2 (4Gy)				
Background	(55.50 ± 10.26) cpm	t_c_ (h)	Mass of sample	R_dry_
Raw meat	(2.98 ± 0.09) kcpm	0:20	3.00 g	
Dried meat	(2.84 ± 0.07) kcpm	0:12	3.00 × (1–0.728)g	72.80%

In experiment 2, no significant differences were measured in the observed data between raw and dried meat samples. A possible reason for the difference in results between experiment 1 and experiment 2 could be due to differences in individual meats. It was confirmed that there were no significant differences in constituent elements of meats for the raw sample and the dried sample in experiment 1 (Supplementary Table 1) with respect to the following elements; Na, Mg, P, K, Ca, Fe, Cu, Zu, Rb. There is a possibility of elements except for the above elements or food additives.

## Discussion

The radioactivation and the modification of proteins and lipids as effects of neutron irradiation on pork shoulder meat are presented in this study. A series of experiments from neutron irradiation experiments to radioactivation and biochemical analysis was carried out.

Fast neutrons are produced by RANS which correspond to the component giving the main contribution as neutron flux in the spacecraft in deep space^7^. The flux in this experiment of order 10^11^ n/cm^2^ is larger than the flux of the 1 MeV neutron inside the spacecraft of order 10^7^ n/cm^2^ in reference^7^. Radioactivation of meat increased with increasing neutron doses. Radioactive nuclei, ^24^Na, ^42^K and ^38^Cl, are shown to be produced in meat by neutron irradiation. Trace elements with a biogenic mass fraction of 1% or less were activated, such as ^23^Na, ^41^K, ^37^Cl and ^42^Ca. Wider research on the activation of food resources by neutrons is important. In order to avoid internal exposure by the radioactivity of food, it is necessary to study the types of food and food additives containing elements causing activation, radiation dose and relaxation time.

The nitrative modification of protein, 6NO_2_Trp, and the oxidative modification of lipid, 4-HNE, in the pork shoulder meat are shown to be caused by the neutron irradiation. In Fig. 5a,b modifications increase as irradiation increase from 0 Gy to around 1 Gy neutron irradiation doses, however around 1 Gy the modification signals saturate or start to decrease. Possible explanations for the saturation or decrease of modification signal above 1 Gy include followings; (1) unknown mechanism of the modification by the neutron irradiation, (2) phenomena caused by insoluble aggregation of proteins, which is likely to occur under conditions of oxidative modification^37^. The correlation between modifications of proteins and lipids with the neutron dose gives clues to the mechanism of modification by neutron irradiation.

The process of modifications of proteins and lipids by neutron irradiation is not understood well yet. The first question is about the neutron reaction with biomolecules of the meat, where two kinds of interactions exist, direct interaction and indirect interaction via water. In our preliminary studies using freeze-dried meat, we obtained essentially the same results on 6NO_2_Trp and 4-HNE formation by using the same WB analyses (data not shown). Therefore, the direct interaction of neutrons with the meat could be the major reaction rather than the indirect interaction through the water. The next question is the mechanism of production of reactive oxygen species (ROS) and nitric oxide radicals (NO), which are required for the formation of 6NO_2_Trp or 4-HNE by the direct interaction. Formation of the oxyradicals by neutron irradiation is supposed to occur as follows: At first, fast neutrons collide with nucleons, protons and neutrons but not electrons, since neutron is electrically neutral. Fast neutrons of a few MeV give energy mainly to hydrogen nuclei by elastic collisions^38^. At this stage, neither electrons nor molecules are irrelevant because the binding energy of the electron (a few keV) is negligible for fast neutrons (a few MeV). Then these knock-on protons lose energy by colliding with electrons in matter. As a result, the atoms are excited or ionized and radicals are produced. This could be the first possible interaction of neutrons with biomolecules. It is necessary to understand which hydrogen of biomolecules or water the neutrons collide with first, and how the electrons left behind after the collision behave.

It is known that neutron causes the deterioration of organic polymers without water^39^. In hydrocarbons fast neutrons interact with hydrogen atoms where the energy deposition is equivalent to the energy of knock-on protons. The knock-on protons interact with electrons causing ionization and excitation of atoms. Along the secondary electron track outside the knock-on proton track, free radicals are formed. Radical migration of active species in paraffin was observed through a crosslinking effect^40^. It is important to investigate a similar process of modifications of the biomolecules.

By the proteome analysis the presence of many oxidative modifications in actin and myosin are found. Approximately 50% of the primary protein supplied by the pork shoulder meat is a component protein of myofibrils. In other words, the effect of neutron irradiation is extremely important when considering oxidative modifications of meat proteins.

In the present experiments, it is shown that the neutron irradiation causes radioactivation of meat and oxidative modifications of proteins and lipids in the meat.　Future work should include similar studies on various food resources and food additives. It is also extremely important to elucidate the interaction between neutrons and biomolecules in order to understand the mechanism of oxidative modification of proteins and lipids by neutron irradiation.

## Methods

### Neutron irradiation—neutron transport simulation with PHITS

The PHITS version 3.15 (Particle and Heavy Ion Transport code System^32^,) code was used to simulate the exposure of the ICRU (International Commission on Radiation Units and Measurements) soft tissue which includes H, C, N and O. Configuration in the target station is as follows: The 7 MeV proton beam bombers the Be target where the meat sample is placed 3 cm separated from the Be target^26^. RANS is usually used to produce thermal neutrons with the moderator^27–31^. However in the present experiment the meat sample was placed at the location of the moderator in such a way that fast neutrons directly irradiate on meat in the sample folder which we had made this time^26^.

### Neutron irradiation—number of neutrons by the indium activation analysis

The neutron flux and the number of neutrons, the number of irradiated neutrons per second per area and the one per area, were obtained by the activation analysis of indium (In). Indium foils were placed with the meat samples under neutron irradiation. The activation analysis utilizes the germanium (Ge) semiconductor detector to count the number of the γ-decay of ^115m^In → ^115^In + γ at the half-life 4.486 h ( the decay constant λ = 4.292 × 10^−5^ s^-1^), emitting the 336.02 keV γ-ray. The radioactive nucleus ^115m^In is generated by the inelastic scattering of neutron and indium, ^115^In(n,n’)^115m^In.

The number of ^115m^In denoted by ${N}_{115m}(t)$ is determined as the sum of the generation of ^115m^In and the contribution of the γ-decays of ^115m^In as

1 $$\frac{{dN_{115m} \left( t \right)}}{dt} = \sigma {\Phi }N_{115} - \lambda N_{115m} \left( t \right),\;N_{115} = \frac{{W\theta N_{A} }}{M}$$

2 $$N_{115m} \left( t \right) = \frac{{\sigma {\Phi }N_{115} }}{\lambda }\left( {1 - e^{ - \lambda t} } \right)$$

where coefficients are given as follows^41^: The cross section of neutron at 1 MeV on ^115^In is $\sigma$ = 0.2 barn^34,35^ (1 barn = 10^–24^ cm^2^). The neutron flux is denoted by $\Phi$ (n/s/cm^2^). The number of ^115^In is ${N}_{115}$. The mass of the indium foil is $W$. The natural abundance of ^115^In is $\theta =$ 0.9571(5). The atomic weight of ^115^In is $M=115$. ${N}_{A}$ is Avogadro constant. The number of ^115m^In becomes $\sigma\Phi {N}_{115}{\lambda }^{-1}(1-{e}^{-\lambda {t}_{i}}) {e}^{-\lambda {t}_{c}}$ after neutron irradiation for the irradiation time ${t}_{i}$ and cooling for the cooling time ${t}_{c}$. The radioactivity of the γ-decay of ^115m^In is given by ${A}_{115m}=\sigma\Phi {N}_{115}(1-{e}^{-\lambda {t}_{i}}) {e}^{-\lambda {t}_{c}}$ ${e}^{-\lambda t}$. For the measurement duration time ${t}_{m}$ the number of the emitted γ-rays is given by its integrant ${\int }_{0}^{{t}_{m}}{dt A}_{115m}$. The number of the counted γ-rays ${C}_{\gamma }$ is obtained ${\int }_{0}^{{t}_{m}}{dt A}_{115m}$ multiplied with the γ-ray branching ratio $\chi =0.4583$ and the detection efficiency $\varepsilon$ which depends on the solid angle of the Ge detector, ${C}_{\gamma }=\chi \varepsilon {\int }_{0}^{{t}_{m}}{dt A}_{115m}$. As a result the neutron flux $\Phi$ is obtained by:

3 $${\Phi } = \displaystyle\frac{\lambda MC_{\gamma }}{ {W\theta N_{A} \sigma \left( {1 - e^{{ - \lambda t_{i} }} } \right)e^{{ - \lambda t_{c} }} \left( {1 - e^{{ - \lambda t_{m} }} } \right)\chi \varepsilon } }$$

The number of neutrons irradiated is a product of the flux and the irradiation time, $N=\Phi {t}_{i}$.

The numbers of neutrons were obtained by the ^115^In activation analysis in Table 1. There is a linear correlation between the number of neutrons and the neutron dose calculated by PHITS (Fig. 2).

### Activation analysis—γ-ray spectrum

The γ-ray spectrum is measured by Ge detector (ORTEC AMETEX-AMT, GMX10P4-70(Ge_2) ). Nuclear databases^33–36^ were used to identify γ-ray spectra and nuclear reactions.

### Biochemical analysis—protein extraction

Prepare protein extracts from neutron-irradiated meat samples. 20 mM Tris–HCl (pH 7.5), 9 M Urea, and 2% CHAPS are used as extraction buffer. After crushing the sample in a bead crusher, centrifuge it (10,000 × g, 10 min, 10 °C) and collect the supernatant. Protein concentration of these extracts was determined by the bicinchoninic acid method (BCA assay reagent; Thermo Fisher Scientific, Rockford, IL, USA) using bovine serum albumin as a standard.

### Biochemical analysis—electrophoresis and WB

For the electrophoresis (SDS-PAGE) 12.5% Polyacrylamide gels are used. Perform a CBB stain to visualize the proteins in the gels. The proteins on the SDS–PAGE gel were transferred on to a PVDF membrane (Immobilon-P, 0.45 mm; Millipore, Billerica, MA, USA) for Western blotting (WB) with a semidry system. The membrane was blocked with 20 ml of 1% bovine serum albumin (BSA) in PBS-T (PBS containing 0.05% Tween20) for 3 hours at room temperature. The membrane was reacted with anti-6-nitrotryptophan (6-NO_2_Trp) monoclonal antibody (10 μg/ml, JaICA, Shizuoka, Japan) or anti-4-hydroxy-2-nonenal (4-HNE) antibody (150 μl/ml, JaICA) in 1%BSA/PBS-T at 4 °C overnight and then treated with alkaline phosphatase-conjugated goat anti-mouse IgG antibody (1/5000, Sigma, STL, USA). The chemiluminescence of the membrane was measured using a LumiCube Plus (Liponics, Inc., Tokyo, Japan) and the signal intensity of bands were analyzed with Image-J software.

### Biochemical analysis—proteome analysis

For the LC–MS/MS analysis, proteins on the SDS–PAGE gel were separated under the same conditions as for Western blotting and stained using Coomassie Brilliant Blue R. The protein bands, which were stained intensively by the immunoreaction, in the sample from neutron-irradiated meat lysates were cut out and digested with trypsin. The tryptic peptides were subjected to LC–MS/MS analysis using a Thermo Fisher Scientific LXQ mass spectrometer (Thermo Fisher Scientifics, MA, USA) with nanoliquid chromatography (AMR, Inc., Tokyo, Japan). The LXQ mass spectrometer system consists of a nanoelectrospray ionization apparatus and anion trap mass spectrometer. The samples were analyzed according to a method described in a previous paper^19^. The conditions of the nano-LC were the following: Zaplous alpha Pep C 18 (0.1 mmi.d. × 150 mm, PEEK coated type, AMR, Inc Tokyo, Japan) and elution with 0.1% formic acid/2% CH_3_CN (solvent A) and 0.1% formic acid / 90% CH_3_CN (solvent B) and flow rate of 500 nl/min. The elution program was the following: after the column equilibration using 5% solvent B, gradient of 2%/min for 30 min. The proteins were identified based on the analysis of the LC–MS/MS data using the MASCOT search engine (Matrix Science, UK) and SwissProt database.

### Comparative experiment—meat samples

The meat samples were taken from a lump of pork shoulder which is composed of protein-rich muscle cells. Pork was the most prominent in the detection of 6nitrotryptophan among pork, beef, fish meat, and sausage^42^. Pork shoulder meats were purchased at butcher stores in Tokyo. We prepared the meat samples with 3 g for the activation analysis and 2 g for the biochemical analysis in the 4 ℃ low-temperature room of Laboratory of Proteomics and Biomolecular Science, Biomedical Research Core Facilities, Juntendo University Graduate School of Medicine. Freeze dryer FTS system FD-3-85D-MP was used to make freeze-dried meat samples.

### Comparative experiment—radiation counts

The radiation count including β-rays and γ-rays is measured by GM counter, Hitachi, Ltd. GM survey meter TGS-1146 with a nominal instrument efficiency of 49.4%, as calibrated with a ^36^Cl β source. The probe is an end-window GM tube that has a thin window of a thickness of ~ 2.5 mg/cm^2^ and a diameter of 5 cm. After the cooling time, radiation counts of meat samples were measured by the GM counter with keeping the positions of the samples.

## Supplementary Information

Supplementary Tables.

Supplementary Figures.

## Data Availability

The datasets used and analyzed during the current study available from the corresponding author on reasonable request.
