# Supplementary material for: Effects of neutron radiation generated in deep space-like environments on food resources
Source: Sci Rep. 2023 Aug 1;13:12479. doi: 10.1038/s41598-023-38990-1 (PMC10394055; doi:10.1038/s41598-023-38990-1)
Supplement: Supplementary file 1 — Supplementary Tables. [file 41598_2023_38990_MOESM1_ESM.pdf]

Supplementary Data

Supplementary Table 1.

Radiation counts of raw and dried meat samples measured by GM survey meter

Experiment 1

	Radiation counts												
	1	2	3	4	5	6	7	8	9	10	Average	SD	unit
Backgound	58	50	43	72							55.75	10.78	cpm
Raw sample No.33	2.34	2.36	2.44	2.5	2.46	2.35	2.41	2.47	2.46	2.52	2.43	0.06	kcpm
Dried sample No.40	8.15	8.16	8.29	8.36	8.41	8.39	8.42	8.46	8.28	8.42	8.33	0.10	kcpm

4.10Gy neutron irradiation

Time of irradiation OFF	15:22	Cooling time	R_{dry}
Backgound	17:11		
Raw sample No.33	17:12	1:50	
Dried sample No.40	17:13	1:51	73.80%

Experiment 2

	Radiation counts												
	1	2	3	4	5	6	7	8	9	10	Average	SD	unit
Backgound	67	53	40	62							55.50	10.26	cpm
Raw sample M7	2.94	2.87	2.95	2.86	2.94	3.02	3.08	3.16	2.97	3.00	2.98	0.09	kcpm
Dried sample M9	2.79	2.80	2.76	2.72	2.79	2.90	2.86	2.92	2.89	2.93	2.84	0.07	kcpm

4.00Gy neutron irradiation

Time of irradiation OFF	12:24	Cooling time	R_{dry}
Backgound	9:20		
Raw sample M7	12:44	0:20	
Dried sample M9	12:36	0:12	72.80%

Supplementary Data

Supplementary Table 2.

Detected elements in the raw and dried meat samples in experiment 1

Detected elements and simplified quantitative values
measured by ICP mass spectroscopy

Samples

1 Raw meat sample of the pork shoulder meat for "raw" sample
2 Dried meat sample of the pork shoulder meat for "dry" sample
3 Raw meat sample of the pork shoulder meat for "dry" sample

Detected chemical element	simplified quantitative values		
	1	2	3
Na	460 ppm	0.15%	520 ppm
Mg	250 ppm	790 ppm	280 ppm
P	0.18%	0.62%	0.19%
K	0.37%	＊1	0.36%
Ca	41 ppm	150 ppm	45 ppm
Fe	5.4 ppm	26 ppm	8.6 ppm
Cu	—	2.2 ppm	—
Zn	21 ppm	73 ppm	24 ppm
Rb	6.6 ppm	23 ppm	7.7 ppm

Japan Food Research Laboratories
