# Supplementary material for: Effects of neutron radiation generated in deep space-like environments on food resources
Source: Sci Rep. 2023 Aug 1;13:12479. doi: 10.1038/s41598-023-38990-1 (PMC10394055; doi:10.1038/s41598-023-38990-1)
Supplement: Supplementary file 2 — Supplementary Figures. [file 41598_2023_38990_MOESM2_ESM.pdf]

Supplemental figure 1: Western blotting using anti nitro-tryptophan antibody

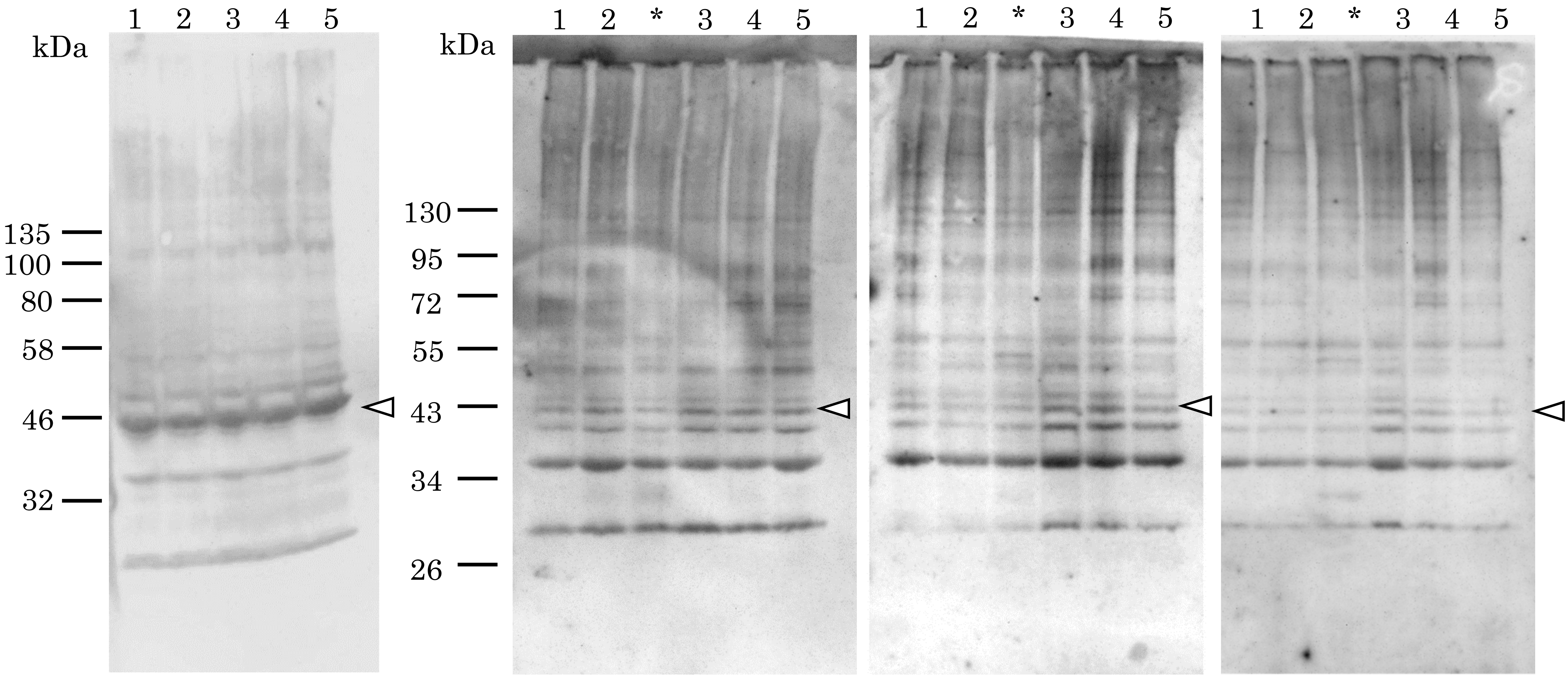

Supplemental figure 1: Western blotting using anti nitro-tryptophan antibody

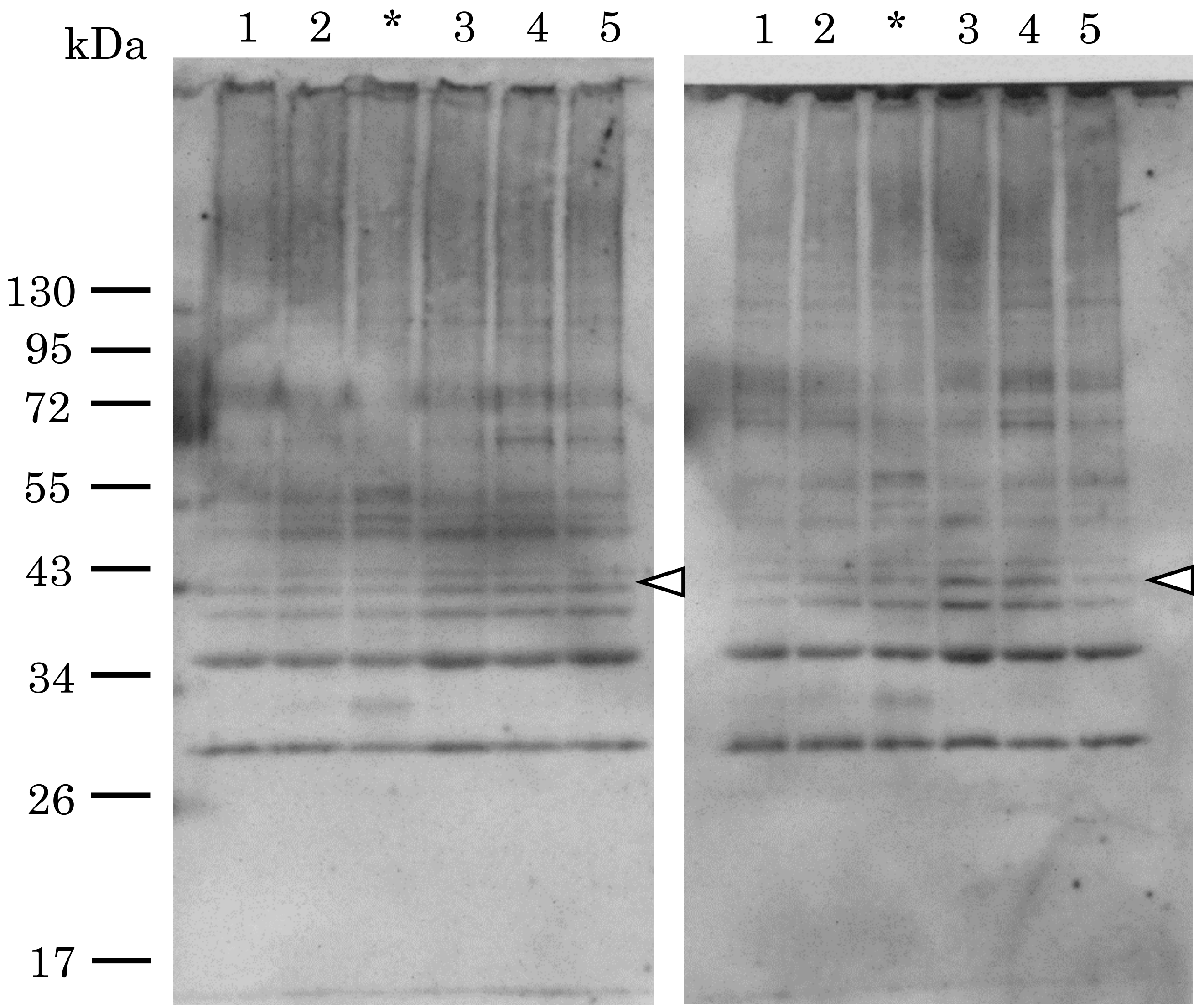

Lane number	Neutron dose
1	0 Gy
2	0.01Gy
* (Pilot experiment)	0.01Gy (Dried meat)
3	0.1 Gy
4	1 Gy
5	3 Gy

Supplemental figure 2: Western blotting using anti 4-HNE antibody

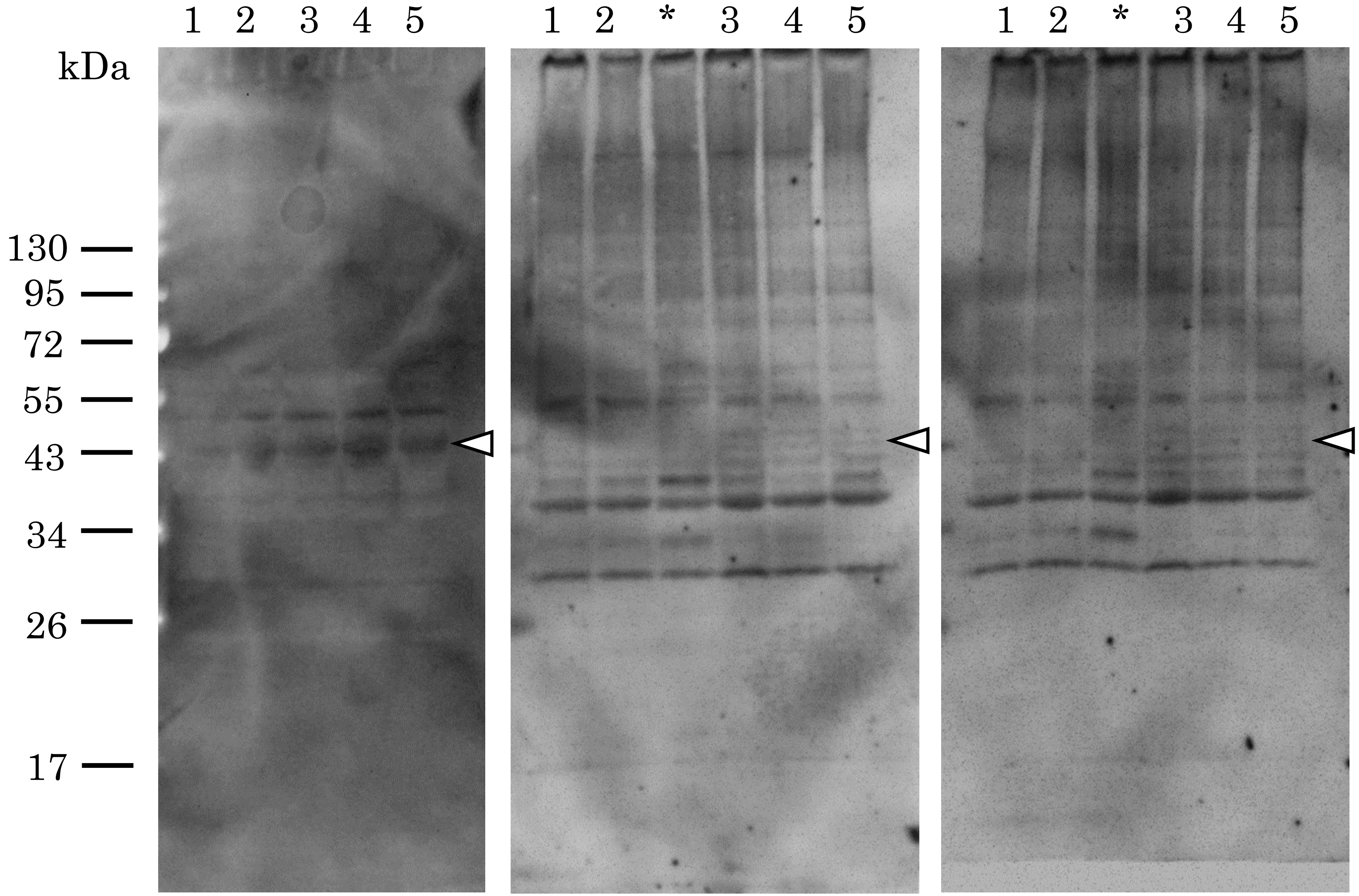

Lane number	Neutron dose
1	0 Gy
2	0.01Gy
* (Pilot experiment)	0.01Gy (Dried meat)
3	0.1 Gy
4	1 Gy
5	3 Gy

Supplemental figure legends

Each Western blot was used to semi-quantify the modifications on the band near the 43kDa. Asterisked lanes are pilot experiments for the dried meat (in this paper, the detailed data of pilot experiments are not shown). The Arrowheads indicate the bands used for the semi-quantify in Fig.5.
